# A novel time-difference electrical impedance tomography algorithm using multi-frequency information

**DOI:** 10.1186/s12938-019-0703-9

**Published:** 2019-07-29

**Authors:** Lu Cao, Haoting Li, Canhua Xu, Meng Dai, Zhenyu Ji, Xuetao Shi, Xiuzhen Dong, Feng Fu, Bin Yang

**Affiliations:** Department of Biomedical Engineering, Air Force Medical University (Fourth Military Medical University), Xi’an, 710032 People’s Republic of China

**Keywords:** Electrical impedance tomography (EIT), Fraction model, Image reconstruction algorithm, Inverse problem

## Abstract

**Background:**

Electrical impedance tomography (EIT) is a noninvasive, radiation-free, and low-cost imaging modality for monitoring the conductivity distribution inside a patient. Nowadays, time-difference EIT (tdEIT) is used extensively as it has fast imaging speed and can reflect the dynamic changes of diseases, which make it attractive for a number of medical applications. Moreover, modeling errors are compensated to some extent by subtraction of voltage measurements collected before and after the change. However, tissue conductivity varies with frequency and tdEIT does not efficiently exploit multi-frequency information as it only uses measurements associated with a single frequency.

**Methods:**

This paper proposes a tdEIT algorithm that imposes spectral constraints on the framework of the linear least squares problem. Simulation and phantom experiments are conducted to compare the proposed spectral constraints algorithm (SC) with the damped least squares algorithm (DLS), which is a stable tdEIT algorithm used in clinical practice. The condition number and rank of the matrices needing inverses are analyzed, and image quality is evaluated using four indexes. The possibility of multi-tissue imaging and the influence of spectral errors are also explored.

**Results:**

Significant performance improvement is achieved by combining multi-frequency and time-difference information. The simulation results show that, in one-step iteration, both algorithms have the same condition number and rank, but SC effectively reduces image noise by 20.25% compared to DLS. In addition, deformation error and position error are reduced by 8.37% and 7.86%, respectively. In two-step iteration, the rank of SC is greatly increased, which suggests that more information is employed in image reconstruction. Image noise is further reduced by an average of 32.58%, and deformation error and position error are also reduced by 20.20% and 31.36%, respectively. The phantom results also indicate that SC has stronger noise suppression and target identification abilities, and this advantage is more obvious with iteration. The results of multi-tissue imaging show that SC has the unique advantage of automatically extracting a single tissue to image.

**Conclusions:**

SC enables tdEIT to utilize multi-frequency information in cases where the spectral constraints are known and then provides higher quality images for applications.

## Background

Electrical impedance tomography (EIT) is a technique in which the electrical properties within a body are imaged using electrical stimulations and measurements applied at electrodes on the body surface. First, forward solutions are computed using the finite-element method to map the association between the impedance inside the body and the boundary voltage outside. Then, the internal impedance image of the object can be reconstructed according to the measured boundary voltage data using an inverse problem algorithm [1]. EIT has advantages such as noninvasiveness, no radiation, and low cost and can be used as a safe and convenient imaging technology in clinical scenarios [2].

Time-difference EIT (tdEIT), which uses single-frequency measurements referred to a baseline, provides images of changes in tissue properties over time [1]. Referring the measured data to the baseline reduces the sensitivity of the method to modeling and instrumentation errors. In tdEIT, the relationship between internal conductivity change and outside voltage change is considered to be linear [2–5]. The linearization is conducted with respect to prior defined conductivity distribution and a solution is usually obtained by solving the regularized linear problem, which results in a short computation time. Thus, it is applicable for real-time monitoring [2]. The overwhelming majority of EIT clinical images are produced using tdEIT; for example, respiration, gastric emptying, and the cardiac cycle [1].

Presently, the various tdEIT algorithms can be categorized into the following approaches: back projection (BP), one-step linear Gauss–Newton (GN), total variation (TV), and GREIT. Their main characteristics are listed in Table 1.

**Table 1:** Comparison of various time-difference algorithms

tdEIT algorithms	Advantages	Disadvantages
BP	Fast imaging speed [6] Easy implementation [7, 24]	Smearing artifacts [9] Low spatial resolution [7, 9]
GN	Fast imaging speed [3] Easy implementation [1, 4] Generally performs better than BP [5]	Target boundary blurred [1]
TV	Sharp edges preserved [12]	Iteration needed [12] Multiple parameters adjustment needed [12]
GREIT	Uniform amplitude response [5]	Training set required [5]
SC	Multi-frequency information integrated Automatic extraction of single tissue Image noise significantly reduced	Tissues conductivity spectra required

1. BP was developed by Barber and Brown in 1984 [6] and is widely used in experimental and clinical EIT [7, 8]. Its original idea is based on an analogy to the algorithm used in CT. With BP, reconstructed images can be simply understood as a superposition of boundary measurements. However, it suffers from low spatial resolution and large smearing artifacts [9] because electric current propagates diffusely and differently to X-ray photons [8].
2. GN has been widely used in EIT since the late 1980s [3, 4]. Its original idea involves transforming the non-linear ill-posed inverse problem into an optimization problem where L2 norm regularization and linearization are used. It meets the needs of real-time imaging and significantly improves image quality compared to BP. Several algorithms with different advantages have been derived by applying the distinct regularization method [5]. One such algorithm is the damped least squares algorithm (DLS), which has been successfully applied in clinical researches with high stability and image quality [9–11].
3. TV is a popular regularization approach that has been applied to a range of imaging modalities [12], with Borsic demonstrating its potential for EIT in 2010 [13]. With TV, sharp discontinuities in images are preserved using L1 norm regularization, which makes its cost function discontinuous and therefore not applicable for GN. Several algorithms have been designed to overcome the non-differentiability of TV and solve it efficiently; for example, Split Bregman (SB) [14], PDIPM [13], and LADMM [15]. However, all three TV methods are iterative and have other parameters that need adjustment, in addition to the regularization parameter [16]. Thus, at present, it may not be available for applications with demand for high temporal resolution such as lung imaging.
4. GREIT was proposed by Adler et al. for lung EIT in 2009 [5]. With the help of a weighting matrix, it integrates the image evaluation process into reconstruction, which results in the solution having more uniform amplitude response and fewer artifacts [16]. The reconstruction matrix is obtained using specific training data that are computed from a forward model. However, an accurate forward model cannot be established in every application scenario. It is also worth noting that when the weighting matrix is uniform, calculation of the reconstruction matrix is equivalent to a scaled generalized Tikhonov solution of GN [5].

All tdEIT algorithms are challenged by the ill-conditioned nature of the inverse problem [4]. One conventional means of overcoming this issue is regularization, which is widely used in the above algorithms. Another means is to increase the amount of data used in one-shot imaging, which is not possible using only measurements at a single frequency [17]. Consider a 16-electrode system for example. Under the opposite-drive adjacent-measurement protocol, the number of dimensions of the data used in an image is limited, 16 × (16 − 4) = 192 (potentials at the injection electrodes are excluded). Multi-frequency measurements cannot be simultaneously employed in traditional tdEIT algorithms because they would lead to an increase in the number of unknowns unexpectedly, because both unknown conductivities and measured voltages vary with frequency [18–20]. Therefore, the idea is to find a parameter that can not only represent tissue distribution in the same manner as conductivity but is also independent of frequency.

Malone et al. [21] recently introduced the volume fraction model into the field of EIT, along with a method for using frequency-difference data in a nonlinear reconstruction scheme by employing spectral constraints. Their method simultaneously uses all multi-frequency measurements to reconstruct the one-shot image because volume fraction is independent of frequency. Studies have shown that fraction imaging can provide satisfactory images when compared to the weighted frequency-difference EIT algorithm [22, 23] on simulated data that violate the assumptions of the latter method. However, this algorithm is an absolute EIT (aEIT) algorithm, which is aimed at providing an absolute distribution of conductivity at a given time. Further, it requires repeated iteration and cannot meet real-time imaging requirements.

Inspired by the study conducted by Malone, this paper proposes a method that reconstructs fraction changes in a linear scheme to realize real-time imaging. In this method, all multi-frequency measurements are directly employed to reconstruct one-shot conductivity change via a frequency-independent fraction model. The proposed method, called the spectral constraints method (SC), successfully incorporates the multi-frequency information into time-difference imaging, and thereby reduces the degrees of freedom and the ill-conditioning of the inverse problem, resulting in improvement of the imaging quality. In this paper, the results of comparison between SC and DLS, made via numerical validation and phantom experiment, are presented. Then, a multi-tissue case and the robustness of SC to spectral errors are discussed. Finally, the principle and advantages of SC are elaborated.

## Results

### Tissue conductivity spectra

The conductivity spectra used for numerical validation and the phantom experiment are shown in Table 2. The measurement results show that the conductivities of the pomelo solution and cucumber increased monotonically from 0.059 and 0.035 S/m at 1 kHz to 0.149 S/m and 0.120 S/m at 200 kHz.

**Table 2:** Conductivity spectra used for numerical validation and phantom experiment

Frequency	Numerical validation (S/m)		Phantom experiment (S/m)	
	Normal brain	Ischemia brain	Pomelo	Cucumber
$\omega_{1}$	0.151	0.115	0.083	0.047
$\omega_{2}$	0.155	0.120	0.105	0.065
$\omega_{3}$	0.175	0.130	0.129	0.093

$\omega_{1}$–$\omega_{3}$ are in the range of 10 Hz–100 kHz

### Numerical validation

**Fig. 1:**
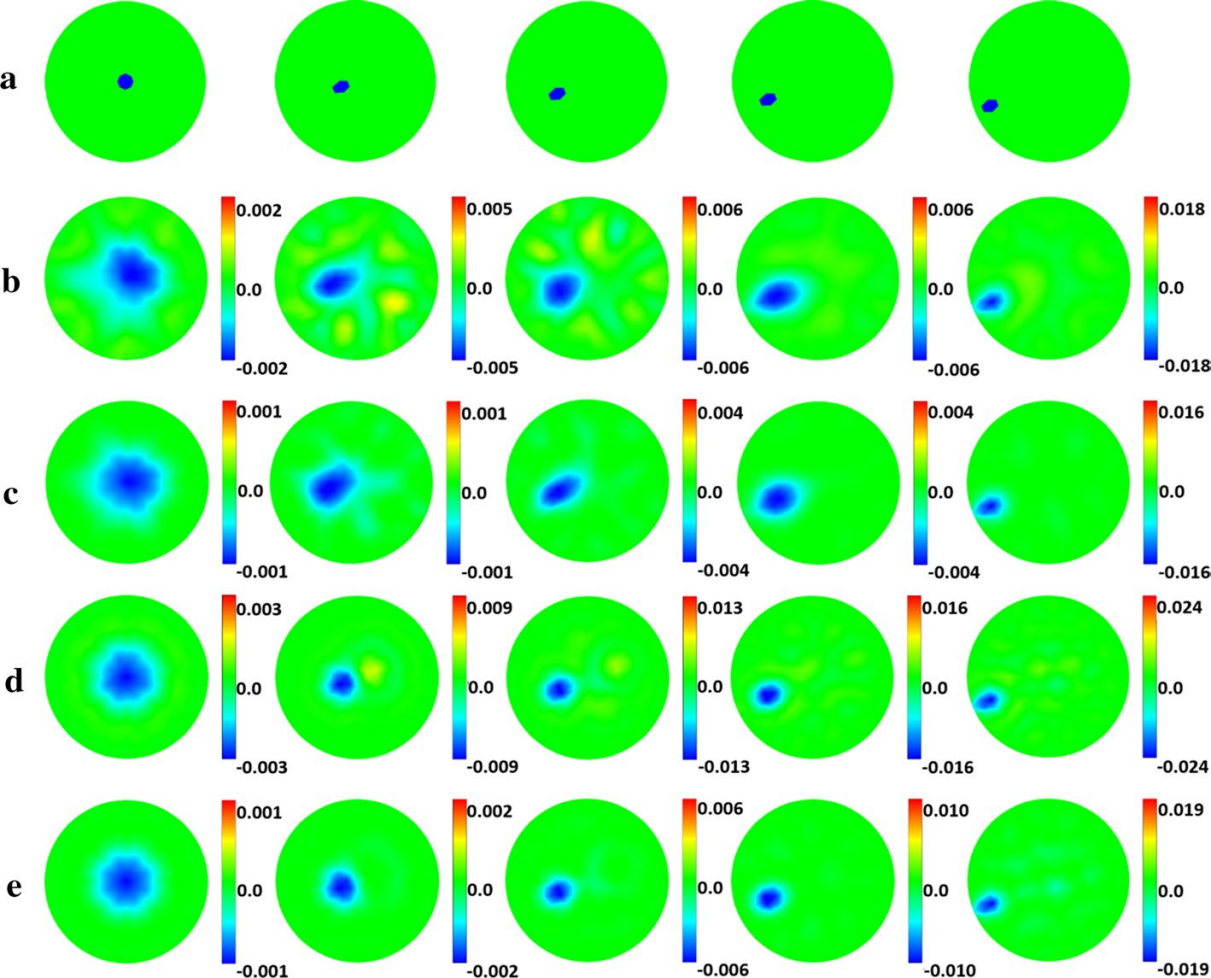
Numerical validation model and results for one-step iteration: **a** Models of five different positions; the order from left to right is Targets 0–4, respectively. Perturbation conductivity images of Targets 0–4 using **b** DLS under SNR = 60 dB, **c** SC under SNR = 60 dB, **d** DLS under SNR = 80 dB, **e** SC under SNR = 80 dB. For each image, the color bar represents the range of the conductivity change, and the numbers represent the maximum range of change. The unit is S/m

The image results of one-step iteration for the two algorithms are shown in Fig. 1b–e. It is evident by visual comparison that the use of SC results in a significant reduction in image artifacts, which is more obvious when signal-to-noise ratio (SNR) is 60 dB and the target is near the center of the imaging domain. Through the analysis in the method section, this can be attributed to the inherent advantage of the fraction model.

**Fig. 2:**
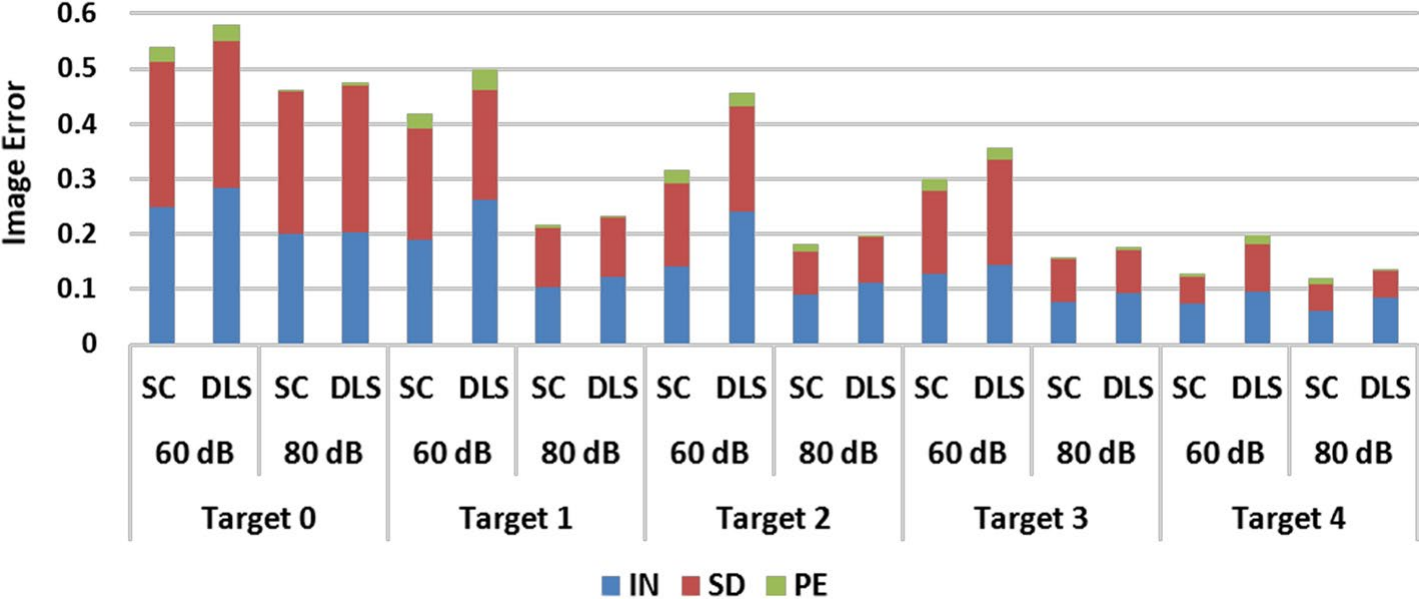
Image quantification results for one-step iteration. (SC: proposed method using spectral constraints; DLS: damped least squares method; IN: image noise; SD: shape deformation; PE: position error)

Next, image quantification and matrix analysis of the two algorithms were conducted. The image quantification results, which are in agreement with visual comparison, are shown in Fig. 2. When SNR is 80 dB, the ${\text{PE}}$ and ${\text{SD }}$ of the two algorithms are similarly small, with the main difference being that SC has a reduced ${\text{IN}}$. When SNR is 60 dB, all three indicators are reduced in SC, especially ${\text{IN}}$. After averaging the data in all target positions and SNR levels, SC reduces ${\text{IN}}$, ${\text{SD}}$, and ${\text{PE}}$ by 20.25%, 8.37%, and 7.86%, respectively, compared to DLS.

The results of matrix $\varvec{S}$ analysis are shown in Table 3. The rank of the two algorithms is exactly the same, which is unexpected. This means that SC increased the number of equations, without increasing the number of independent equations. The reason for this is that $\varvec{S}$ is a partitioned matrix obtained by longitudinal stitching. According to the rank characteristics of the partitioned matrix, if the ranks of all element matrices are equal and the distributions of the independent columns are uniform, the rank of the whole matrix is equal to the rank of the element matrix, ${\text{Rank}}\left( \varvec{S} \right)\, = \,{\text{Rank}}\left( {\varvec{J}\left( {\omega_{i} } \right)\varvec{A}\left( {\omega_{i} } \right)} \right)$. Further, as $\varvec{A}\left( {\omega_{i} } \right)$ is a full rank matrix, ${\text{Rank}}\left( \varvec{S} \right)\, = \,{\text{Rank}}\left( {\varvec{J}\left( {\omega_{i} } \right)\varvec{A}\left( {\omega_{i} } \right)} \right)\, = \,{\text{Rank}}\left( {\varvec{J}\left( {\omega_{i} } \right)} \right)$. Thus, there is no significant difference between the two algorithms as regards rank. As for condition number, the slight difference in that order of magnitude between the two algorithms is also of no significance. Except for Target 0 under 60 dB, SC has a smaller condition number.

**Table 3:** Matrix $\varvec{S}$ indexes of the two algorithms under one-step iteration in numerical validation

SNR	Index	SC					DSL				
		Target 0	Target 1	Target 2	Target 3	Target 4	Target 0	Target 1	Target 2	Target 3	Target 4
60 dB	Rank	76	76	76	76	76	76	76	76	76	76
	Cond	87.88	87.88	87.88	87.88	87.88	94.87	84.64	84.64	84.64	84.64
80 dB	Rank	76	76	76	76	76	76	76	76	76	76
	Cond	87.88	87.88	87.88	87.88	87.88	84.64	84.64	84.64	84.64	84.64

**Fig. 3:**
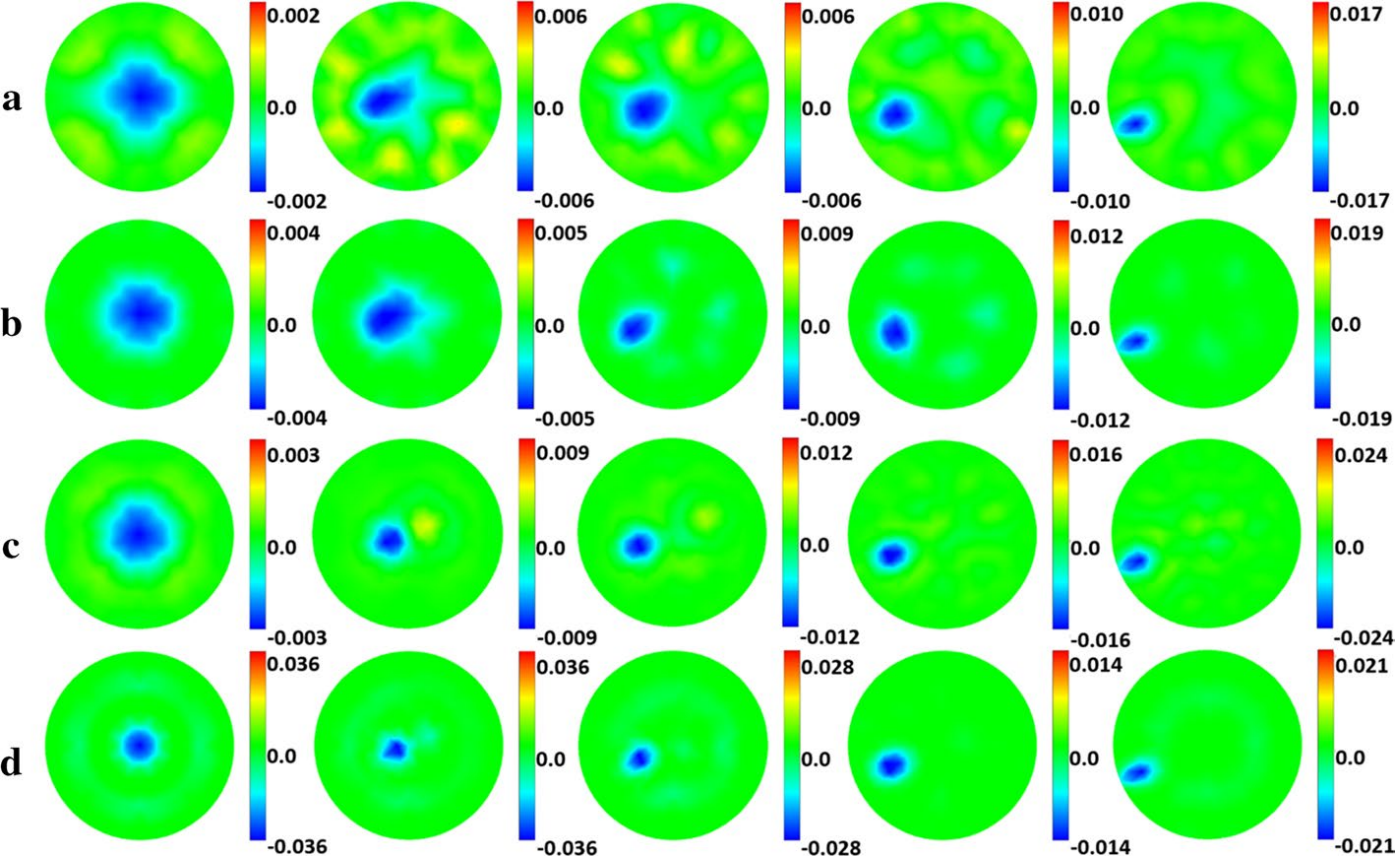
Image results for two-step iteration: Perturbation conductivity images of Targets 0–4 using **a** DLS under SNR = 60 dB, **b** SC under SNR = 60 dB, **c** DLS under SNR = 80 dB, **d** SC under SNR = 80 dB. For each image, the color bar represents the range of the conductivity change, and the numbers represent the maximum range of change. The unit is S/m

Based on the above analysis, it can be concluded that the rank of $\varvec{S}$ can be effectively increased as long as the independent column distribution of matrix $\varvec{ J}\left( {\omega_{i} } \right)$ changes with frequency, which will happen when tissue distribution in the background frame is uneven. As we all know, with iteration, the tissue distribution in the background frame becomes uneven. Therefore, two-step iteration was further performed with both algorithms to determine whether SC can reduce the degrees of freedom and ill-conditioning of the inverse problem. The image results of two-step iteration are shown in Fig. 3a–d. The artifacts are significantly reduced, which was already observed in one-step iteration. In addition, the shape deformation is reduced especially when target is near the center and SNR is 80 dB, which were not perceived in the one-step iteration.

**Fig. 4:**
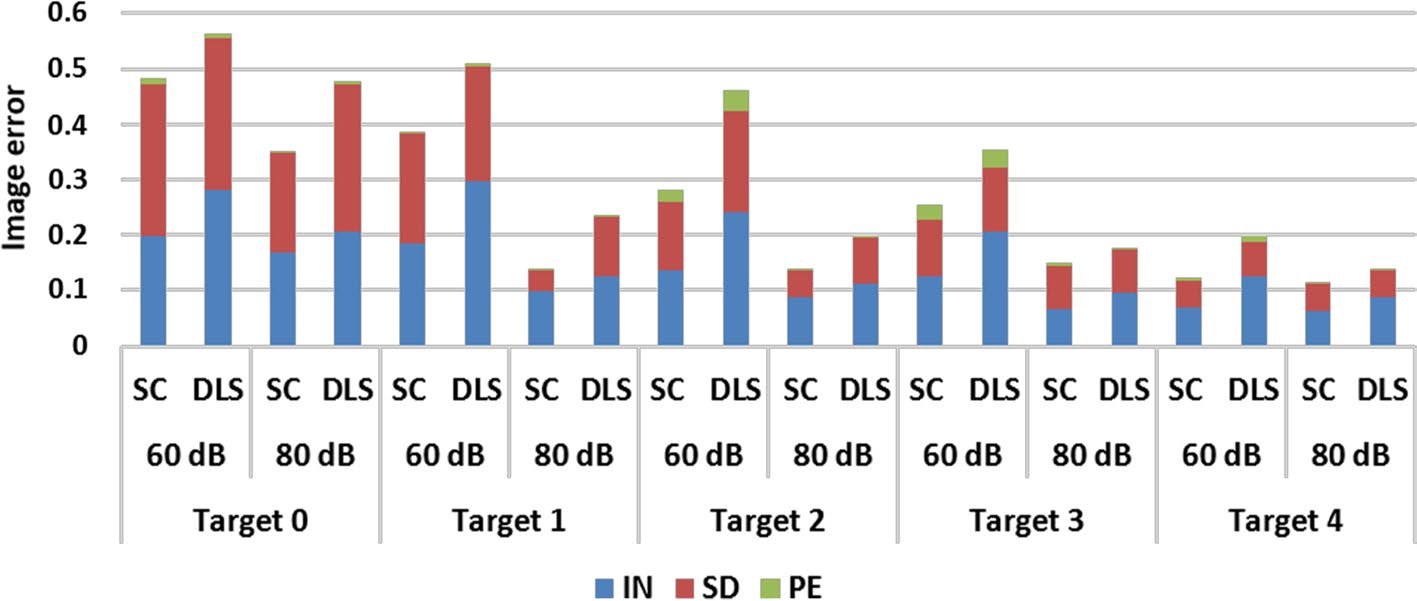
Image quantification results for two-step iteration. (SC: proposed method using spectral constraints; DLS: damped least squares method; IN: image noise; SD: shape deformation; PE: position error)

The image quantification results under two-step iteration are shown in Fig. 4. When SNR is 80 dB, the ${\text{PE}}$ of the two algorithms is very small, and the main difference is that SC has smaller ${\text{IN}}$ and ${\text{SD}}$, especially for Targets 1 and 2. When SNR is 60 dB, all three indicators of SC are smaller than those of DLS. On average, SC reduces ${\text{IN}}$, ${\text{SD}}$, and ${\text{PE}}$ by 32.58%, 20.20%, and 31.36%, respectively. Compared to the results in one-step iteration, all indicators are further reduced, especially ${\text{SD}}$ and ${\text{PE}}$, which corroborates our previous conjecture.

Table 4 shows the result of matrix $\varvec{S}$ analysis under two-step iteration. The condition numbers of SC decreased for Targets 0–2 and increased for Targets 3–4 when compared to DLS. However, the rank of SC increased significantly for all targets, as expected. This means that SC reduced the degrees of freedom of the inverse problem.

**Table 4:** Matrix $\varvec{S}$ indexes of the two algorithms under two-step iteration in numerical validation

SNR	Index	SC					DSL				
		Target 0	Target 1	Target 2	Target 3	Target 4	Target 0	Target 1	Target 2	Target 3	Target 4
60 dB	Rank	118	128	132	125	133	76	76	76	76	76
	Cond	78.70	97.99	85.45	118.17	107.05	97.85	137.39	99.39	89.42	90.53
80 dB	Rank	120	126	130	132	136	76	76	76	76	76
	Cond	79.36	86.13	91.31	124.93	95.25	107.12	91.80	91.80	94.44	89.18

**Fig. 5:**
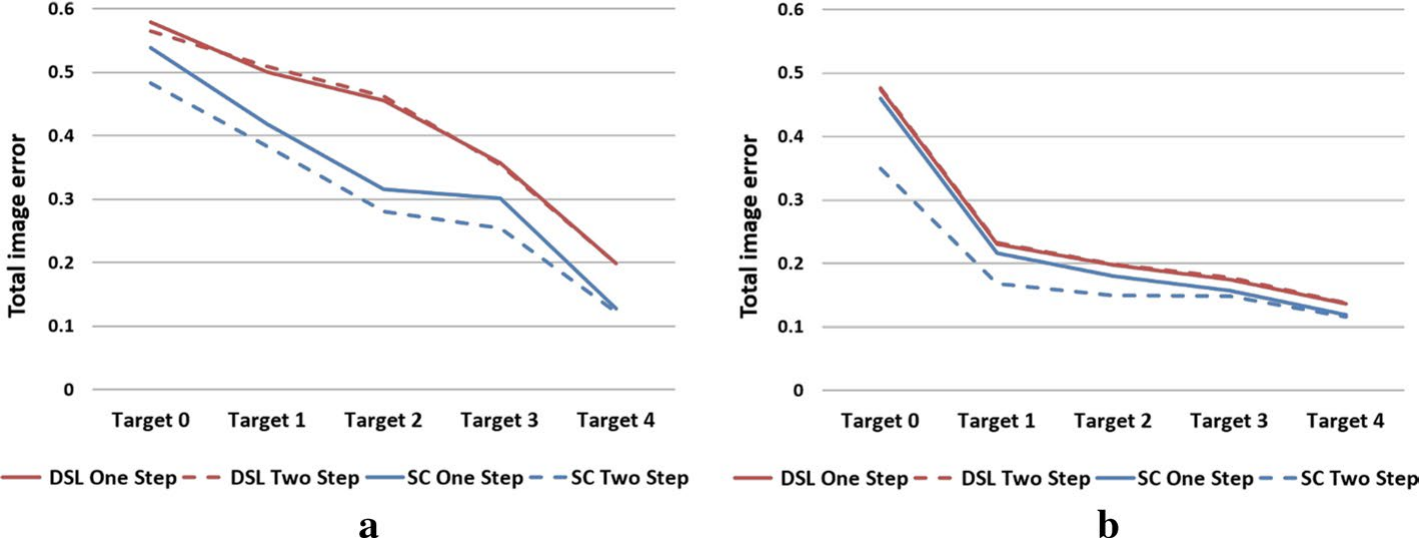
Comparison of total image error under two SNR levels: **a** SNR = 60 dB, **b** SNR = 80 dB

To further illustrate the image effect of reducing the degrees of freedom of the inverse problem, the ${\text{TE}}$ of the two algorithms was compared. The results in Fig. 5 show that: (1) with or without iteration, SC has a smaller ${\text{TE}}$ than DLS, and the gap between them is more obvious when SNR is 60 dB; (2) SC further reduced ${\text{TE}}$ in the case of two-step iteration, and this reduction is more obvious when the target is near the center; however, DLS slightly increased ${\text{TE}}$ in some locations, which we assume is mainly caused by modeling errors. Then, by averaging the data, SC reduces ${\text{TE}}$ by 12.16% under one-step iteration and 28.14% under two-step iteration. These illustrate that, with iteration of limited steps, SC can further improve the image quality by reducing the degrees of freedom of the inverse problem. This is a unique advantage that DLS does not have.

### Phantom study

**Fig. 6:**
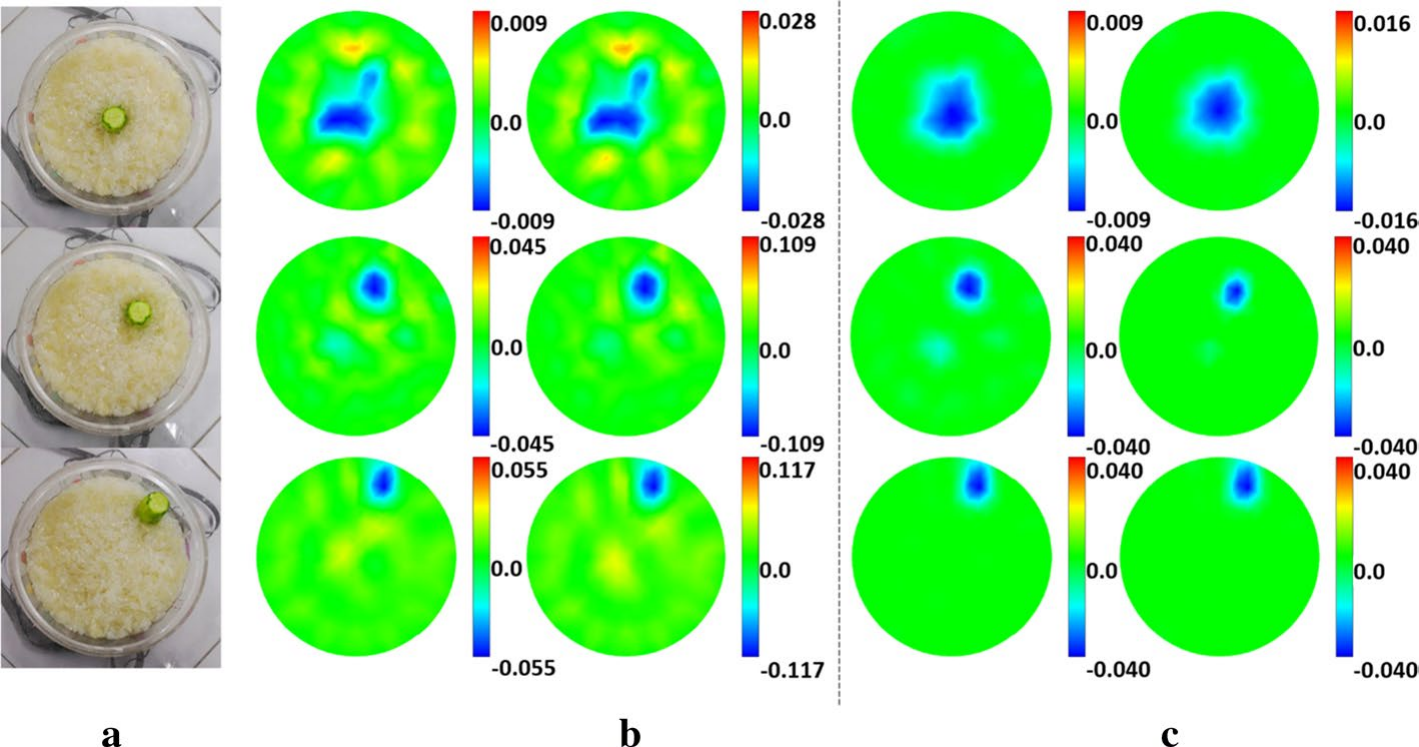
Phantom experiment reconstructed images of the two algorithms: **a** real target setup. **b**, **c** Reconstructed images of **b** the DLS algorithm and **c** the proposed SC algorithm under one-step and two-step iteration. For each image, the color bar represents the range of the conductivity change, and the numbers represent the maximum range of change. The unit is S/m

Figure 6 shows the results of the phantom experiment. SC effectively suppresses image artifacts and has higher edge-preservation ability. In particular, when the target is close to the center, DLS fails, while SC performs well. Moreover, the image quality is further improved when SC is iterated in two steps, but no improvement for DLS is perceived.

The results of matrix $\varvec{S}$ analysis are shown in Table 5. It can be seen that SC effectively increased the rank and decreased the condition number of $\varvec{S}$ when iterated in two steps, which means that it reduced the degrees of freedom and the morbidity of the inverse problem.

**Table 5:** Matrix $\varvec{S}$ indexes of the two algorithms in the phantom study

Iteration number	Index	SC			DLS		
		Target 0	Target 1	Target 2	Target 0	Target 1	Target 2
One	Rank	76	76	76	76	76	76
	Cond	84.62	84.62	84.62	84.64	84.64	84.64
Two	Rank	144	165	160	76	76	76
	Cond	79.35	99.94	82.47	109.84	102.75	115.04

**Fig. 7:**
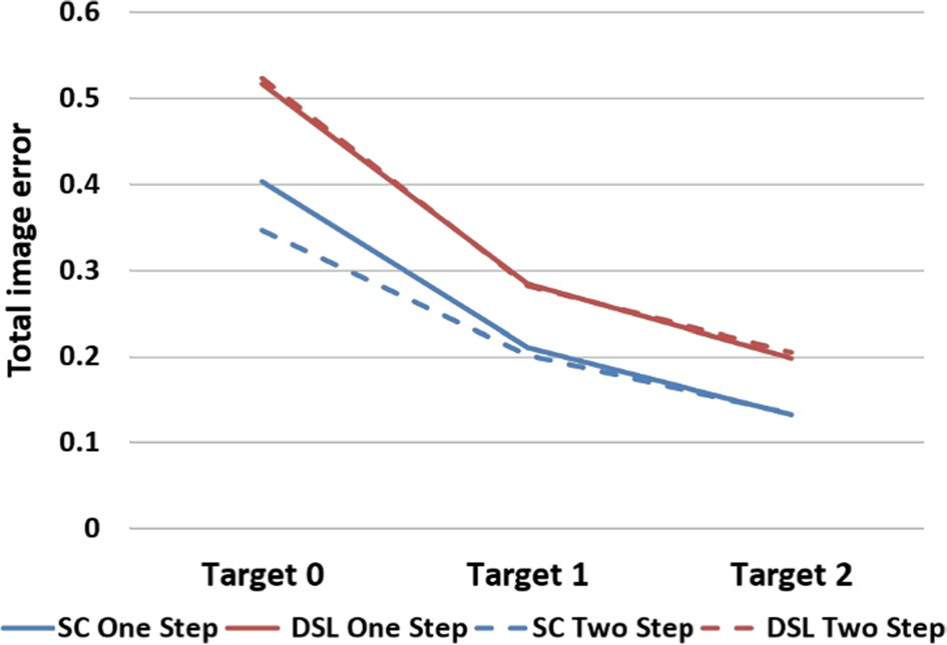
Phantom experiment evaluation results

The results of comparison of ${\text{TE}}$ between the two algorithms are shown in Fig. 7. The results show the same tendency as compared with the data acquired from the numerical validation. Overall, SC reduces ${\text{TE}}$ by 25.75% under one-step iteration and 36.54% under two-step iteration.

### Robustness to spectral errors

Accurate conductivity spectra are fundamental to SC. However, errors exist in the spectra data obtained from both methods described in the experimental setup section [25]. To study the influence of spectral errors, a simulation study was performed. First, the mean relative contrast between the tissues was defined [21]:

1 $$C^{\% } = \frac{1}{M}\mathop \sum \limits_{i = 1}^{M} \left( {\frac{{\varepsilon_{i2} - \varepsilon_{i1} }}{{\varepsilon_{i1} }}} \right)$$

For the brain tissue spectrum used in the simulation, $C = 24.05\%$.

**Fig. 8:**
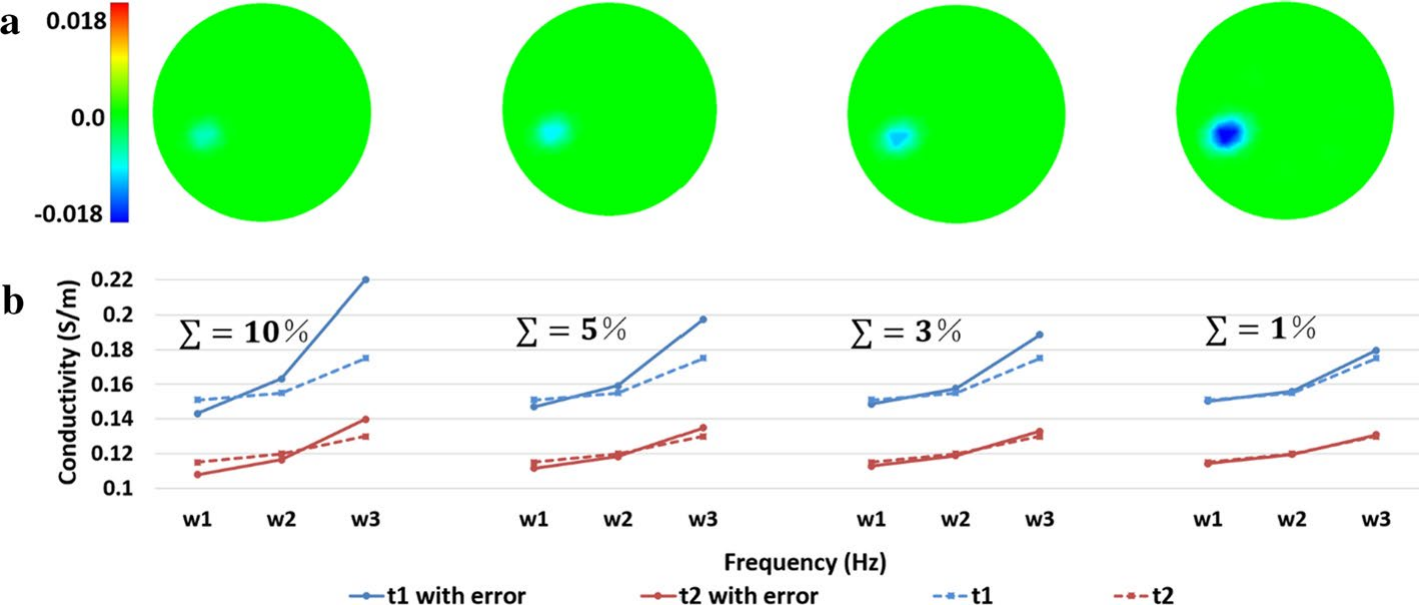
Robustness to spectral errors results: **a** reconstructed images for each value of $\sum$. **b** Comparison between the original spectrum and the spectrum with errors. The color bar represents the range of the conductivity change, and the numbers represent the maximum range of change. The unit is S/m

**Fig. 9:**
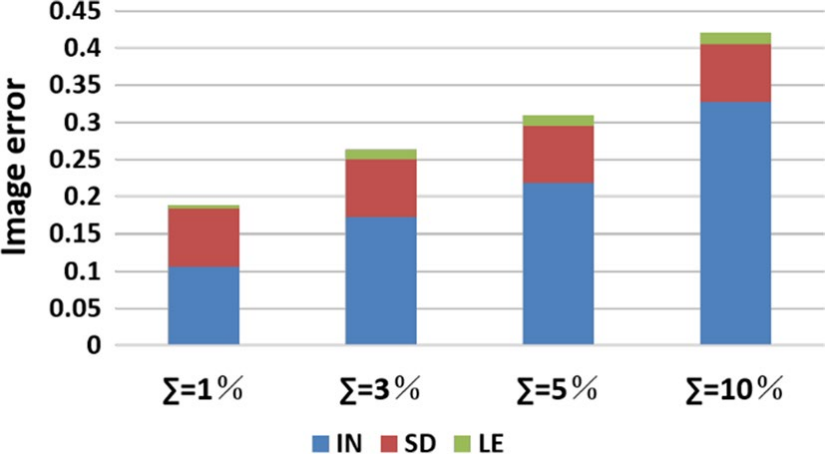
Image quantification results for each value of $\sum$. (IN: image noise; SD: shape deformation; PE: position error)

Then, a random error was added to the conductivity spectra of normal brain tissue $\varepsilon_{1}$ and ischemic brain tissue $\varepsilon_{2}$ before producing a conductivity model:

2 $$\sigma_{\text{ni}}^{*} = \left\{ {\begin{array}{*{20}c} {\varepsilon_{i1} \, + \,{\text{Rand}}\,\left( {0,\,\varepsilon_{i1} *\sum } \right)\,{\text{on}}\, {\text{the}}\, {\text{background}}} \\ {\varepsilon_{i2} \, + \,{\text{Rand}}\,\left( {0,\,\varepsilon_{i2} *\sum } \right)\,{\text{on }}\,{\text{the}}\, {\text{pertubation}}} \\ \end{array} } \right.$$

where $\sum \, = \,1\% , \,3\% ,\,5\% , \,{\text{and}} \,10\%$ were, respectively, selected, $i$ is the frequency index and ${\text{Rand}}\left( {0,\,\varepsilon_{ij} *\sum } \right)$ is a random number drawn from the normal distribution with mean zero and variance $\varepsilon_{i1} *\sum$. Finally, the boundary voltage data were simulated using the original model, and fraction images were reconstructed using the model $\varvec{\sigma}^{*}$. The reconstruction results and comparison between the original spectrum and the spectrum with errors are shown in Fig. 8. The evaluation results are shown in Fig. 9. The results show that image errors increased with the spectral errors, while the amplitude response decreased. In particular, when the spectral error was 5% and 10%, the target response was weak and difficult to distinguish by the human eye.

### Multiple tissue case

One promising application of SC is tissue identification. As the tissues were classified before imaging, following reconstruction, the target images of each tissue were easily obtained without extra steps. However, only two tissues and one target were set in the study presented above, which aimed at drawing a comparison between SC and DLS. To further verify the feasibility of SC in the multi-tissue case and demonstrate its tissue identification capability, it was applied to a preliminary numerical validation with multiple tissues.

The number of tissues was increased to three to generate simulation data among which $t_{3}$ blood was added. The conductivity spectrum of the blood remains unchanged at 0.7 S/m over a wide range. Thus, the blood conductivity at the three chosen frequencies is 0.7 S/m.

**Fig. 10:**
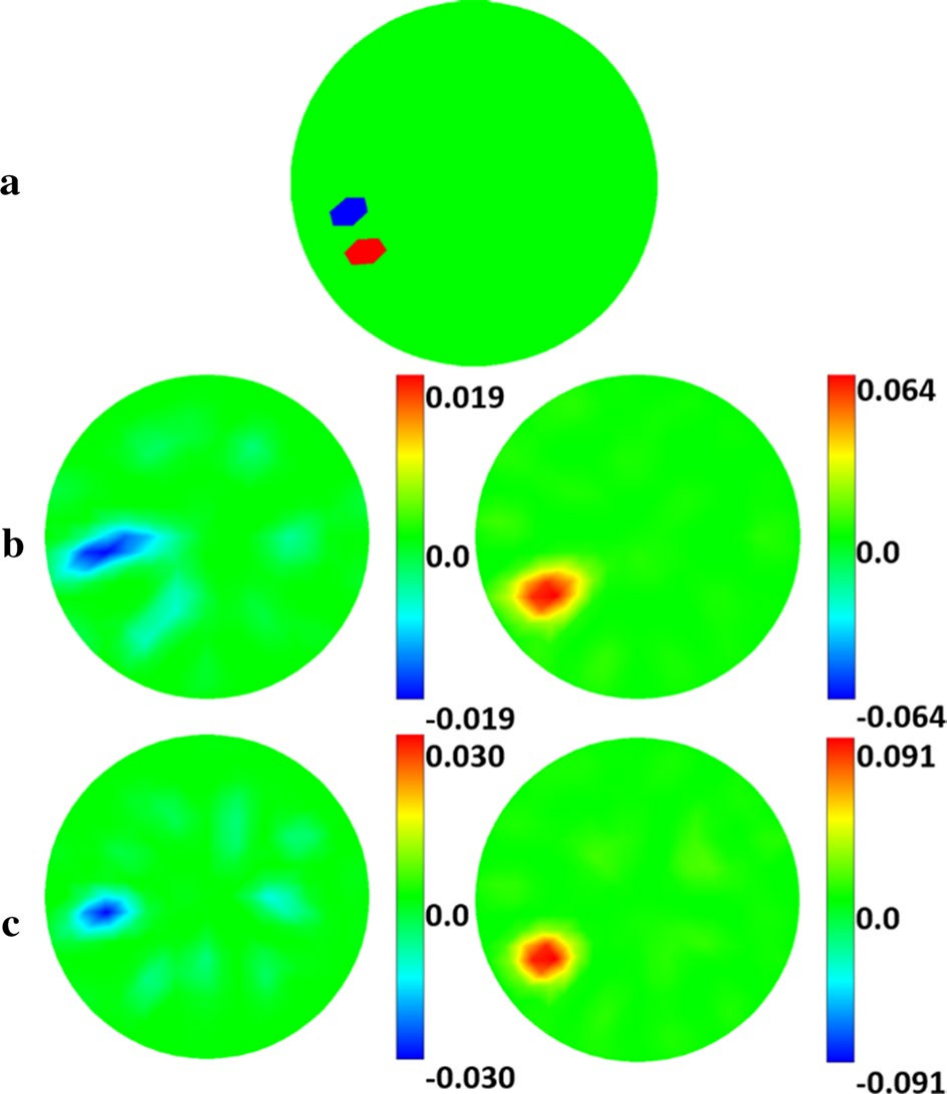
Three-tissue case model and reconstruction: **a** numerical model. Reconstructed images of different interested tissues under **b** SNR = 60 dB, **c** SNR = 80 dB. For each image, the color bar represents the range of the conductivity change, and the numbers represent the maximum range of change. The unit is S/m

The reconstruction process was fundamentally the same as that of the two-tissue experiment. However, at the end of the reconstruction, the tissue of interest could be easily extracted by fraction value to image. The results of two-step iteration at two SNR levels are shown in Fig. 10. The target images of hemorrhagic and ischemic tissue were easily obtained, respectively. However, the image quality of the ischemic target was found to be inferior to that of the hemorrhagic target, especially for SNR = 60 dB.

## Discussion

In this study, we introduced and verified a novel tdEIT algorithm that employs fraction model and spectral constraints. We applied the proposed SC algorithm to synthetic and measured data and found that it significantly reduced artifacts and shape deformation errors compared to the DLS algorithm. We also demonstrated that this advantage increased with the iteration step. The reasons why SC effectively improves imaging quality are as follows:

1. SC reconstructs a parameter that does not vary with frequency. The number of unknowns depends only on the number of tissues and elements, which is constant. Thus, we can reconstruct one frame time-difference image by simultaneously using data at multiple frequencies. By iteration, this method effectively reduces the degrees of freedom and the morbidity of the reconstruction problem, and improves the image quality.
2. SC limits the fraction value to the range [0, 1]. As a result, the conductivity in each element is also limited to a certain range, and thus some artifacts are removed. This is the reason why even without iteration, the image quality is still improved.

In this study, only data under three different frequency points were used, because for our reconstruction model three is the minimum to make the number of equations greater than the number of unknowns. However, the results show that $\varvec{S}$ is not full rank after two-step iteration and continuing iteration would marginally increase rank (less than five) but significantly increase computation time. It is necessary to further explore the variation of the image quality with the frequency number and thereby standardize the frequency selection method.

The results also show that, under two-step iteration, the rank of $\varvec{S}$ increased in both the numerical simulation and the phantom experiment; however, the condition number differed. For Targets 0–2, it decreased in both experimental methods, whereas for Targets 3–4, it unexpectedly increased only in the numerical simulation. The following reasons could account for this. First, the target position and tissue spectra of the two experiments were not exactly the same. Second, the properties of $\varvec{S}$ can be affected by the iteration number; therefore, iteration was continued to three steps in the simulation experiment. The results show that the rank continued to increase marginally and the condition number decreased for all target positions, especially for Targets 3–4, where the condition number was less than that obtained in one-step iteration. However, the reconstruction images under three-step iteration were not presented because they were similar to those of two-step iteration and the number of iteration steps should be limited within two to meet the requirement of 1 frame in 1 s.

As for the multi-tissue case, the image quality of the ischemic target was found to be inferior to that of the hemorrhagic target. One of the main reasons for this is that, within the selected frequency band, the conductivity changes of hemorrhagic tissue relative to normal tissue are much larger than those of ischemic tissue. This situation can be improved by optimizing the selection of frequency points. However, as the blood spectrum remains unchanged over a wide frequency range, the corresponding improvement will be limited. Therefore, further studies are necessary to determine how image quality varies with the number of tissues and how to obtain optimal multi-target imaging results. As regards spectral errors, the results reveal that the image quality decreased with increasing spectral errors. Therefore, further studies are necessary to improve the anti-spectral error performance of SC. For this problem, Malone et al. previously proposed a reconstruction classification algorithm [26] that can reconstruct the image and estimate the tissue spectrum simultaneously. However, further investigation is needed to determine whether this method can be successfully applied to tdEIT.

## Conclusions

In this study, we developed a novel tdEIT algorithm (called SC) to obtain enhanced reconstructed images by exploiting multi-frequency information. Specifically, we redesigned the existing tdEIT framework by introducing the concept of volume fraction to enable simultaneous utilization of multi-frequency data to reconstruct one frame image.

The results of numerical and phantom experiments verify that SC has superior performance in reducing image error, especially image noise, when compared with the DLS algorithm. The results also reveal a unique advantage of SC, which is that it can effectively reduce the degrees of freedom and morbidity of the inverse problem and further improve the image quality when it iterates in limited steps.

Since the imaging results of each tissue are easily screened by the fraction model, further work will be conducted to develop the potential for multi-target identification. In addition, imaging quality is affected by spectral errors and frequency number; further studies will be needed to reduce the influence of spectral errors and standardize the frequency selection method. Overall, SC may provide a new direction for the development of tdEIT algorithms in cases where the spectral constraints are known.

## Methods

### Fraction model

A fraction model is a representation of the tissue distribution within an object [21, 27]. It assumes the object consists of a limited number of tissues. After meshing the object, volume fractions or concentration values of different tissues can be determined for each element. Meanwhile, if the conductivity spectra of all tissues are known, the conductivity distribution is also obtained by transforming the fraction model.

The model can be summarized as follows.

1. The domain is composed of *T* kinds of distinct tissues, $t_{1} \ldots t_{j} \ldots t_{T}$.
2. The impedance spectra of each tissue are known. For example, the conductivity of the $j{\text{th}}$ tissue for the $i{\text{th}}$ frequency is $\sigma_{ij} = \varepsilon^{{t_{j} }} \left( {\omega_{i} } \right)$.
3. The conductivity of the $n{\text{th}}$ element is equal to the linear weighted sum of the conductivities of the component tissues [28].

3 $$\sigma_{n} \left( {\omega_{i} } \right) = \mathop \sum \limits_{j = 1}^{T} f_{nj} *\sigma_{ij}$$

where $0\, \le \,f_{nj} \, \le \,1\, {\text{and }}\,\mathop \sum \nolimits_{j = 1}^{T} f_{nj} \, = \,1$. The weighting value $f_{nj}$ is the volume fraction of the $j{\text{th}}$ tissue in the $n{\text{th}}$ element.

Let us take a two-dimensional region ${\varvec{\Omega}}$ that is composed of *T* kinds of tissues as an example. First, discretization of the domain is performed using the finite-element method (FEM), and $N$ is the number of elements. As the object contains $T$ kinds of tissues, there is a fraction value $f_{nj}$ in each element for every tissue. Subsequently, the fraction matrix $\varvec{F}_{\text{matrix}} \in \varvec{R}^{T*N}$ is obtained, among which the $n{\text{th}}$ column is the proportion of each tissue inside the $n{\text{th}}$ element. Then, the matrix is vectorized by column to obtain the fraction vector, $\varvec{F} = \varvec{vec}\left( {\varvec{F}_{\text{matrix}} } \right),\varvec{F} \in \varvec{R}^{{\left( {T*N} \right)*1}}$:


Next, the element-based and piecewise constant conductivity distribution is approximated using the fraction model. The conductivity vector can then be represented as $\varvec{\sigma}\left( {\omega_{i} } \right) \in \varvec{R}^{N*1}$. Finally, currents are injected at the boundary of $M$ frequencies ($\omega_{1} \ldots \omega_{i} \ldots \omega_{M}$), and for each frequency a boundary voltage vector $\varvec{\upsilon}\left( {\omega_{i} } \right) \in \varvec{R}^{K *1}$ is acquired at a given time, where $K$ is the number of measurements. By comparing this vector with the background frame, a boundary voltage difference vector $\Delta\varvec{\upsilon}\left( {\omega_{i} } \right)$ is obtained. The whole difference vector $\Delta\varvec{\upsilon}$ can be obtained by longitudinally stitching the $M$ vectors ($\Delta\varvec{\upsilon}\left( {\omega_{1} } \right) \cdots \Delta\varvec{\upsilon}\left( {\omega_{i} } \right) \cdots \Delta\varvec{\upsilon}\left( {\omega_{M} } \right)$).

### Forward problem

Based on the fraction model described above, the discrete forward problem is deduced as follows:

First, the linear relationship between fraction value and conductivity is obtained according to assumption (3):

4 $$\varvec{A}\left( {\omega_{i} } \right)\varvec{F} =\varvec{\sigma}\left( {\omega_{i} } \right)$$

where $\varvec{A}\left( {\omega_{i} } \right) \in \varvec{R}^{{N*\left( {T*N} \right)}}$ is a coefficient matrix formed by the conductivity spectra of $T$ kinds of tissues:

$$A(\omega_{i} ) = \left[ {\begin{array}{*{20}c} {\varepsilon^{{t_{1} }} \left( {\omega_{i} } \right)} & {\varepsilon^{{t_{2} }} \left( {\omega_{i} } \right)} & \cdots & {\varepsilon^{{t_{\text{T}} }} \left( {\omega_{i} } \right)} & 0 & 0 & \cdots & 0 & \cdots & 0 & 0 & \cdots & 0 \\ 0 & 0 & \cdots & 0 & {\varepsilon^{{t_{1} }} \left( {\omega_{i} } \right)} & {\varepsilon^{{t_{2} }} \left( {\omega_{i} } \right)} & \cdots & {\varepsilon^{{t_{\text{T}} }} \left( {\omega_{i} } \right)} & \cdots & 0 & 0 & \cdots & 0 \\ \vdots & \vdots & \ddots & \vdots & \vdots & \vdots & \ddots & \vdots & \ddots & \vdots & \vdots & \ddots & \vdots \\ 0 & 0 & \cdots & 0 & 0 & 0 & \cdots & 0 & \cdots & {\varepsilon^{{t_{1} }} \left( {\omega_{i} } \right)} & {\varepsilon^{{t_{2} }} \left( {\omega_{i} } \right)} & \cdots & {\varepsilon^{{t_{\text{T}} }} \left( {\omega_{i} } \right)} \\ \end{array} } \right]$$

Second, in the traditional tdEIT algorithm, the discrete forward problem without noise is as follows [29]:

5 $$\varvec{J}\left( {\omega_{i} } \right)\Delta\varvec{\sigma}\left( {\omega_{i} } \right) = \Delta\varvec{\upsilon}\left( {\omega_{i} } \right)$$

where the Jacobian matrix $\varvec{J}\left( {\omega_{i} } \right) \in \varvec{R}^{K*N}$ is the first derivative of $\varvec{\upsilon}\left( {\omega_{i} } \right)$ with respect to $\varvec{\sigma}\left( {\omega_{i} } \right),$ and can be calculated using the standard derivation method or the compensation theorem method [1, 4].

By substituting Eq. (4) into Eq. (5), the ideal discrete forward problem of fraction change reconstruction is obtained:

6 $$\varvec{J}\left( {\omega_{i} } \right)\varvec{A}\left( {\omega_{i} } \right)\Delta \varvec{F} = \Delta\varvec{\upsilon}\left( {\omega_{i} } \right)$$

where both $\varvec{J}$ and $\varvec{A}$ vary with frequency. Contrarily, $\Delta \varvec{F}$ is independent of frequency, which makes it possible to simultaneously employ multi-frequency measurements to reconstruct one-frame image by viewing $\Delta \varvec{F}$ as the unknown parameter.

### Inverse problem

To simultaneously utilize the multi-frequency information, the following objective function is constructed to minimize the norm of the data mismatch under all frequencies:

7 $${\varvec{\Phi}} = \frac{1}{2}||\varvec{S}\Delta \varvec{F} - \Delta\varvec{\upsilon}||^{2}$$

$\varvec{S}$ is an assembly matrix that includes $\varvec{M}$ element matrices, each of which is obtained by multiplying the $\varvec{J}$ and $\varvec{A}$ under the same frequency:

8 $$\varvec{S} = \left[ {\begin{array}{*{20}c} {J\left( {\omega_{1} } \right)A\left( {\omega_{1} } \right)} \\ {J\left( {\omega_{2} } \right)A\left( {\omega_{2} } \right)} \\ { \ldots \ldots } \\ {J\left( {\omega_{M} } \right)A\left( {\omega_{M} } \right)} \\ \end{array} } \right]$$

The objective function can also be written in an equivalent form [30] (Eq. 9):

9 $${\varvec{\Phi}} = \frac{1}{2}\mathop \sum \limits_{i = 1}^{M}|| \varvec{J}\left( {\omega_{i} } \right)\varvec{A}\left( {\omega_{i} } \right)\Delta \varvec{F} - \Delta\varvec{\upsilon}\left( {\omega_{i} }\right)||^{2}$$

Using the standard Tikhonov regularization method [3], the objective function becomes as follows:

10 $${\varvec{\Phi}} = \frac{1}{2}\left[||{\varvec{S}\Delta \varvec{F} - \Delta\varvec{\upsilon}||^{2} + \lambda ||\varvec{R}\Delta \varvec{F}||^{2} } \right]$$

The regularization parameter $\lambda$ controls the trade-off between fidelity and robustness [31, 32]. The L-curve method is chosen for both SC and DLS to determine the regularization parameter $\lambda$ because it can be implemented efficiently and easily [33–35]. $\varvec{R}$ is the regularization matrix and represents the inverse of the covariance of the expected image. The Standard Tikhonov method is chosen for both SC and DLS, which sets $\varvec{R} = {\text{diag}}\left( {\varvec{S}^{T} \varvec{S}} \right)$ in SC and $\varvec{R} = {\text{diag}}\left( {\varvec{J}^{T} \varvec{J}} \right)$ in DLS.

To satisfy the constraint $\mathop \sum \nolimits_{j = 1}^{T} f_{nj} = 1 \forall n$, a substitution is made in the objective function ($f_{n1} = 1 - \mathop \sum \nolimits_{j = 2}^{T} f_{nj}$). Then, the unknown parameter becomes $\Delta \varvec{F}_{{\varvec{T} - 1}}$ ($t_{2} \ldots t_{j} \ldots t_{T}$). Correspondingly, matrix $\varvec{S}$ will change to $\varvec{S}^{\prime}$. The final objective is as follows:

11 $${\mathbf{\varPhi^{\prime}}} = \frac{1}{2}\left[ {\varvec{S}^{\prime}\Delta \varvec{F}_{{{\text{T}} - 1}} - \Delta\varvec{\upsilon}^{2} + \lambda \varvec{R}\Delta \varvec{F}_{{{\text{T}} - 1}}^{2} } \right]$$

The solution is obtained by setting the first derivative of ${\varvec{\Phi}} '$ to zero [36]:

12 $$\Delta \varvec{F}_{{T - 1}} = \left( {\varvec{{S}^{\prime}}^{T} {\text{*}}\varvec{S}^{\prime} + \lambda \varvec{R}} \right)^{{ - 1}} {\text{*}}\varvec{{S}^{\prime}}^{T} {\text{*}}\Delta \varvec{\upsilon }$$

The temporary fraction vector $\tilde{\varvec{F}}$ of $\varvec{T}$ tissues is obtained by combining initial fraction distribution $\varvec{F}^{0}$ and $\Delta \varvec{F}_{T - 1}$. The final $\varvec{F}$ is obtained by adding the constraints of Eq. (13) to $\tilde{\varvec{F}}$:

13 $$f_{\text{ni}} \, = \,\left\{ \begin{aligned} & 0\quad {\text{if}}\;\widetilde{{f_{{{\text{ni}}}} }} \le 0 \\ & 1\quad {\text{if}}\;\widetilde{{f_{{{\text{ni}}}} }} \ge 1 \\ & \widetilde{{f_{{{\text{ni}}}} }} \quad {\text{otherwise}} \\ \end{aligned} \right.$$

The fraction change $\Delta \varvec{F}$ is derived from Eq. (14):

14 $$\Delta \varvec{F} = \varvec{ F} - \varvec{F}^{0}$$

Under the condition of satisfying a certain imaging speed, the performance of SC can be improved by linear iteration of limited steps (${\text{NUM}}$). Then, the reconstruction process is summarized as follows:

Step 0: Initialize fraction vector $\varvec{F}^{0}$ and the number of iteration steps to $k = 1$
Step 1: If $k \le {\text{NUM}},$ repeat Steps 2–5; otherwise, output $\Delta \varvec{F}$
Step 2: Set the first derivative of the objective function to zero and get $\Delta \varvec{F}_{T - 1}^{k}$
Step 3: Combine $\varvec{F}^{k - 1}$ and $\Delta \varvec{F}_{T - 1}^{k}$ to get $\tilde{\varvec{F}}^{k}$
Step 4: Impose the constraints in Eq. (13) on $\tilde{\varvec{F}}^{k}$ to get $\varvec{F}^{k}$ and update the objective function and $\Delta\varvec{\upsilon}$
Step 5: Set $\Delta \varvec{F} = \varvec{F}^{k} - \varvec{F}^{0}$ and $k = k + 1$; return to Step 1

Finally, to compare with DLS, the final fraction change needs to be transformed into conductivity change using the linear relationship in Eq. (4).

### Matrix $\varvec{S}$ evaluation

In this section, the reasons why SC can improve image quality are theorized and two indicators of $\varvec{S}$ are proposed to verify them.

First, SC needs to impose the constraints in Eq. (13) on the calculated solution $\tilde{\varvec{F}}$. According to the linear relationship in Eq. (4), the corresponding conductivity will also be limited. This inherent characteristic of SC limits image artifacts to a certain extent and improves image quality.

Second, SC simultaneously uses multi-frequency data to reconstruct one shot image. This is in direct contrast to the DLS, which uses the data of only one frequency. The number of equations is $M$ times that of the DLS algorithm; that is, $M*K$. The unknowns are a multiple of $\left( {T - 1} \right);$ that is, $\left( {T - 1} \right)*N$. When $M*K \ge \left( {T - 1} \right)*N$ is satisfied, SC is expected to increase the number of independent equations, reduce the degrees of freedom and morbidity of the inverse problem, and improve image quality. To verify this conjecture, the following two indicators are proposed for $\varvec{S}$, which is inversed in the reconstruction process [37]:

#### Rank

Rank corresponds to the maximum number of linearly independent columns or rows of a matrix. It is thus a measure of the “nondegenerateness” of the system encoded by this matrix. There are numerous equivalent solutions for rank. In this study, the Singular Value Decomposition (SVD) method is adopted.

#### Condition number

In the field of numerical analysis, the condition number of a matrix signifies how much the output vector of the system encoded by this matrix can change for a small change in the input vector. A problem with a high condition number is said to be ill-conditioned [36], which means that a small amount of noise at its input will have a significant impact on its output. It is defined as the ratio between the maximum and the minimum singular value of this matrix.

### Image evaluation

We considered the case of two tissues and one target occupied by $t_{2}$, while the background is occupied by $t_{1}$. In accordance with the objective quantification method proposed by Adler [5], four indicators—position error, deformation error, image noise, and total image error—are used to evaluate the performance of the two algorithm. First, the region of perturbation (RP) is defined, which satisfies the following two conditions [21]:

- Values larger than 50% of the maximum displacement from the mean value of the image [21].
- The largest connected cluster of voxels in (a).

#### Position error (PE)

PE is defined as the ratio of the displacement distance of the centroid of the reconstructed perturbation from the real position to the diameter of the mesh $d^{\text{MESH}}$:

15 $${\text{PE}} = \frac{{\left| {d^{\text{RP}} - d^{\text{REAL}} } \right|}}{{d^{\text{MESH}} }}$$

where $d^{\text{RP}}$ and $d^{\text{REAL}}$, respectively, represent the distance from the centroid of the reconstructed perturbation and that of the real perturbation to the center of the model.

#### Deformation error (DE)

DE is defined as the averaged difference in *X*–*Y* dimensions between the real and reconstructed perturbations.

16 $${\text{SD}} = \frac{1}{2}\left( {\frac{{\left| {l_{x}^{\text{RP}} - l_{x}^{\text{REAL}} } \right| + \left| {l_{y}^{\text{RP}} - l_{y}^{\text{REAL}} } \right|}}{{d^{\text{MESH}} }}} \right)$$

Among them, $\left( {l_{x}^{\text{RP}} ,l_{y}^{\text{RP}} } \right)$ and $\left( {l_{x}^{\text{REAL}} ,l_{y}^{\text{REAL}} } \right)$, respectively, represent the width on the *X*–*Y* dimensions of the reconstructed and real perturbations.

#### Image noise (IN)

IN is defined as the inverse of the contrast-to-noise ratio (CNR) between the real perturbation and the background:

17 $${\text{IN}} = \frac{{\sqrt {\frac{1}{{N^{B} - 1}}\mathop \sum \nolimits_{n \in B} \left( {\Delta f_{n2} - \Delta \tilde{f}_{2}^{B} } \right)^{2} } }}{{\left| {\Delta \tilde{f}_{2}^{\text{REAL}} - \Delta \tilde{f}_{2}^{B} } \right|}}$$

where $\Delta \tilde{f}_{2}^{\text{REAL}}$ and $\Delta \tilde{f}_{2}^{B}$ are the mean fraction change of the real perturbation and the background, and $N^{B}$ is the number of elements of the background.

#### Total image error (TE)

Overall, the error of an image is described by adding the three errors described above:

18 $${\text{TE}}\,{ = }\,{\text{PE}}\,{ + }\,{\text{SD}}\,{ + }\,{\text{IN}}$$

### Experimental setup

#### Tissue impedance spectra

In numerical validation, the conductivity spectra of human brain tissues taken from the literature were used [38]. The background and perturbation conductivities were set to the values for normal brain and ischemic brain.

In the phantom experiment, fruit and vegetable objects with frequency-dependent conductivities were used to mimic the properties of live tissues. The background medium was a mixture of 0.1% NaCl solution and pomelo granules, and the perturbation was a cucumber segment of diameter 2.5 cm and height 7 cm. Conductivity measurements were acquired with a Solartron 1294 impedance analyzer for 25 frequencies in the range 1–200 kHz using Ag–AgCl electrodes. This frequency range is consistent with our imaging system, FMMU-EIT5 [39]. The cucumber was cut into 3 mm × 3 mm cubes for placement in the measurement box, which had a diameter of 1 cm and a height of 1.2 cm and was connected to the analyzer via a conductor. Three different samples were selected for each tissue, and measurements were made five times for each sample at room temperature. The conductivity was calculated using the specific calculation formula for this measurement box, and the final conductivity spectra were obtained by averaging.

Three frequency points were intercepted from the broad spectra obtained via the two different methods mentioned above to generate the final spectra, because when $M = 3$ the number of equations is greater than the number of unknowns for our reconstruction model.

#### Design of the numerical validation

**Fig. 11:**
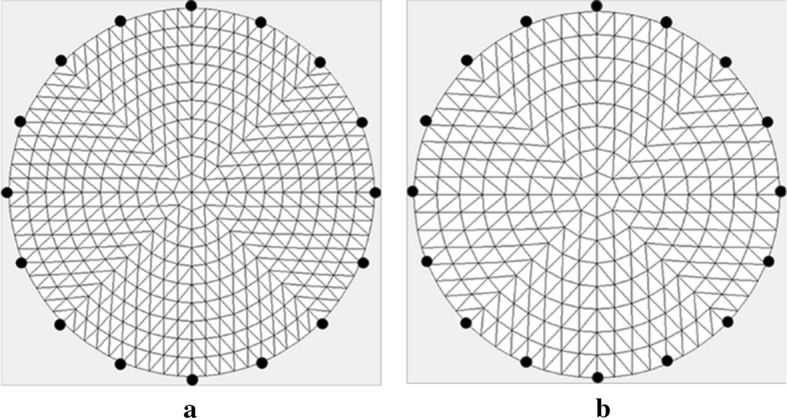
Numerical simulation in a circular mesh model: **a** forward model with 800 elements and 441 nodes. **b** Inverse model with 512 elements and 289 nodes

In the numerical validation, SC and DLS were applied to synthetic data, and the reconstructed images were compared. The generation of synthetic data is based on a circular mesh model with a radius of 300 pixels divided into 800 elements and 441 nodes. Further, 16 electrodes are evenly placed on the edge (Fig. 11a). A current of peak amplitude 1 mA was injected into two electrodes placed polarly, and the difference between the voltages on all adjacent pairs of electrodes not involved in delivering the current was measured, for a total of 192 measurements per frequency. The ground point was fixed at the center of the mesh. After generating the boundary voltage data, two levels of Gaussian noise were added, with SNRs of 60 dB and 80 dB, respectively.

An elliptical perturbation with an average length of six pixels was set at five different locations (Fig. 1a). The disturbance centers were 0, 60, 120, 180, and 240 pixels from the center of the circle. The conductivity of the background and perturbation at different frequencies were set to the values shown in Table 2.

Each target generated two frames of measurement data, namely, background frame without target and foreground frame with target. Then, SC and DLS were applied to reconstruct the images. The reconstruction was based on a circular mesh model divided into 512 elements and 289 nodes, which differed from the generation model to avoid the inverse crime (Fig. 11b). The regularization parameter was selected via the L-curve method and the two indexes of matrix $\varvec{S}$ were calculated simultaneously in the reconstruction process.

#### Design of the phantom study

A phantom study was designed to reproduce the experimental setup introduced previously in the simulation. The boundary voltage data were generated across an acrylic tank filled with biological tissues. The tank was 17 cm in diameter and 7 cm in height, and 16 electrodes are evenly placed on its edge.

**Fig. 12:**
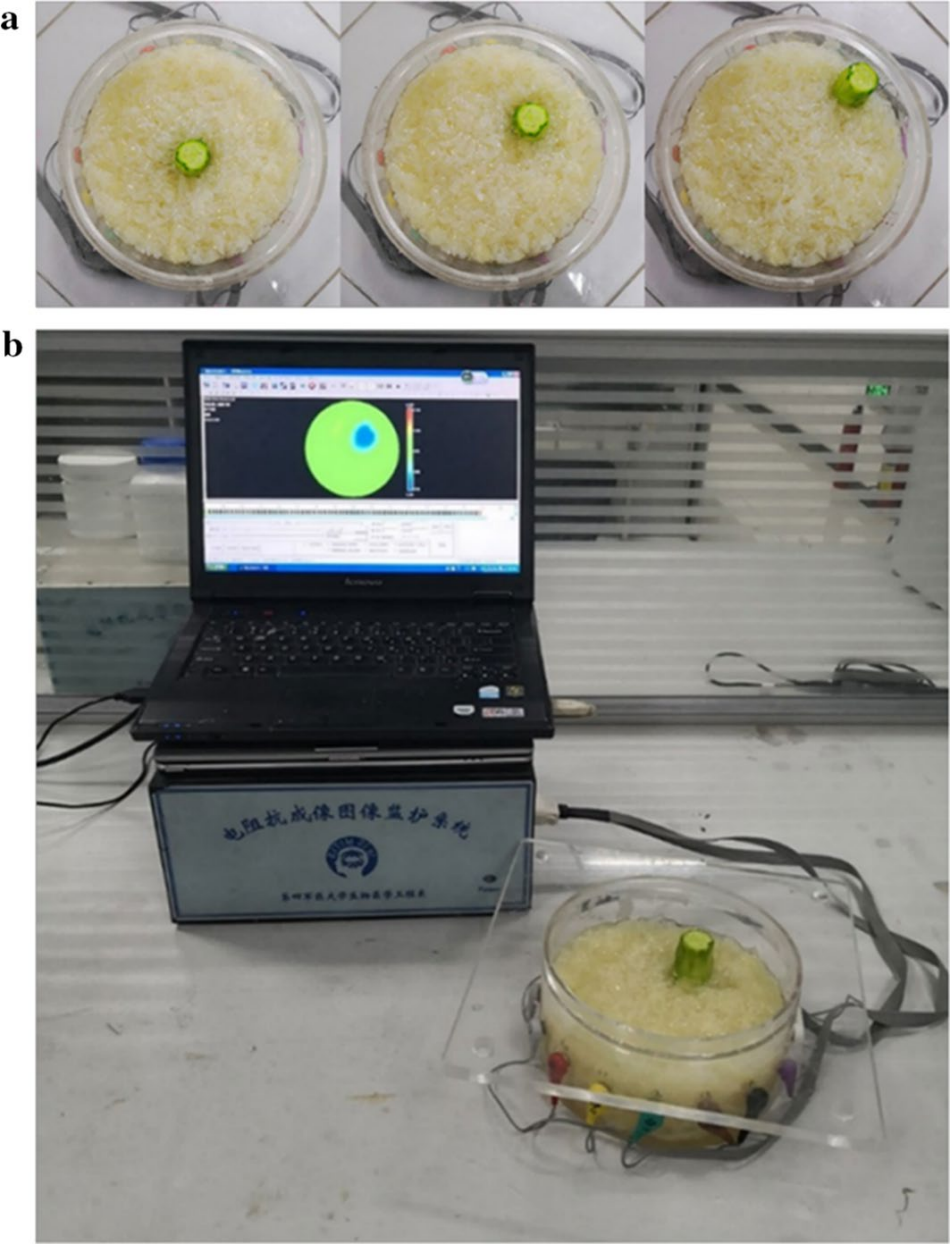
Phantom experiment setup and models: **a** perturbation locations; the order from left to right is Targets 0–2. **b** Data acquisition system

The cucumber was placed in three different positions, as shown in Fig. 12a, and immersed in the saline–pomelo mixture. The three perturbations, Targets 0–2, were 0 cm, 4.25 cm, and 6.5 cm away from the center of the tank, respectively.

Data acquisition was conducted using the FMMU-EIT5 system [39] shown in Fig. 12b. This EIT data acquisition system can produce a programmable current with SNR greater than 89 dB and can also measure the voltage difference precisely with CMRR higher than 75 dB [39]. The three excitation frequencies were set at 20 kHz, 50 kHz, and 100 kHz. The peak amplitude of the injected current was set at 1 mA. The used background frame and the foreground frame were averaged over 10 frames, respectively. Images were reconstructed using the same mesh employed in numerical validation. In the following, unless otherwise specified, the regularization parameter was selected using the L-curve method.

In addition to the experiments above, which were designed to compare the performance of SC and DLS, another two preliminary simulation experiments were conducted to explore the possibility of multi-tissue imaging and the influence of spectral errors. The specific implementation plans were presented in the results section.

## Data Availability

The data sets used during the current study are available from the corresponding author on reasonable request.
